# Recalcitrant cell wall of *Ulva lactuca* seaweed is degraded by a single ulvan lyase from family 25 of polysaccharide lyases

**DOI:** 10.1016/j.aninu.2022.01.004

**Published:** 2022-02-05

**Authors:** Mónica M. Costa, Luís B. Pio, Pedro Bule, Vânia A. Cardoso, Marlene Duarte, Cristina M. Alfaia, Diogo F. Coelho, Joana A. Brás, Carlos M.G.A. Fontes, José A.M. Prates

**Affiliations:** a CIISA - Centro de Investigação Interdisciplinar em Sanidade Animal, Faculdade de Medicina Veterinária, Universidade de Lisboa, Alto da Ajuda, 1300-477 Lisboa, Portugal; b NZYTech - Genes and Enzymes, Estrada do Paço do Lumiar, Campus do Lumiar, Edifício E, 1649-038 Lisboa, Portugal

**Keywords:** Macroalga, *Ulva lactuca*, Carbohydrate-active enzyme, Ulvan lyase, Cell wall degradation

## Abstract

Green macroalgae, e.g., *Ulva lactuca*, are valuable bioactive sources of nutrients; but algae recalcitrant cell walls, composed of a complex cross-linked matrix of polysaccharides, can compromise their utilization as feedstuffs for monogastric animals. This study aimed to evaluate the ability of pre-selected Carbohydrate-Active enZymes (CAZymes) and sulfatases to degrade *U. lactuca* cell walls and release nutritive compounds. A databank of 199 recombinant CAZymes and sulfatases was tested in vitro for their action towards *U. lactuca* cell wall polysaccharides. The enzymes were incubated with the macroalga, either alone or in combination, to release reducing sugars and decrease fluorescence intensity of Calcofluor White stained cell walls. The individual action of a polysaccharide lyase family 25 (PL25), an ulvan lyase, was shown to be the most efficient in cell wall disruption. The ulvan lyase treatment, in triplicate measures, promoted the release of 4.54 g/L (*P* < 0.001) reducing sugars, a mono- and oligosaccharides release of 11.4 and 11.2 mmol/100 g of dried alga (*P* < 0.01), respectively, and a decrease of 41.7% (*P* < 0.001) in cell wall fluorescence, in comparison to control. The ability of ulvan lyase treatment to promote the release of nutritional compounds from alga biomass was also evaluated. A release of some monounsaturated fatty acids was observed, particularly the health beneficial 18:1c9 (*P* < 0.001). However, no significant release of total fatty acids (*P* > 0.05), proteins (*P* = 0.861) or pigments (*P* > 0.05) was found. These results highlight the capacity of a single recombinant ulvan lyase (PL25 family) to incompletely disrupt *U. lactuca* cell walls. This enzyme could enhance the bioaccessibility of *U. lactuca* bioactive products with promising utilization in the feed industry.

## 1 Introduction

Over the years, the exploitation of macroalgae for food, feed and biotechnological industries has been gathering increasing attention (Makkar et al., 2016). This is due to the presence of bioactive and nutritive compounds, which makes them a valuable resource (Makkar et al., 2016). Carbohydrates constitute a large portion of macroalgae biomass (up to 76%) (Rioux and Turgeon, 2015), whereas lipids only range from 1.5% to 6.6% in *Ulva* sp. green seaweed (Costa et al., 2021). Nevertheless, green macroalgae may present considerable amounts of health-promoting (Givens, 2009) monounsaturated fatty acids (MUFA) and polyunsaturated fatty acids (PUFA) (Cardoso et al., 2017; Neto et al., 2018). However, protein content is very variable, with values ranging between 8.65% (Mæhre et al., 2014) and 31.6% of the total biomass in *Ulva lactuca* (Ripol et al., 2018).

Green seaweeds (*Chlorophyceae*) belong mostly to the genus *Ulva* and present a cell wall composed of an intricate carbohydrate matrix (Popper et al., 2011). The main polysaccharides that constitute green algae cell walls are soluble ulvan and insoluble cellulose, as well as trace amounts of alkali-soluble xyloglucan and glucuronans (Lahaye and Robic, 2007). In addition, mannose and galactose were also reported in some alga species, with arabinose having been described in *U. lactuca*. Ulvan is a branched and gel-forming polysaccharide that comprises 8% to 29% dry wt of algae biomass (Lahaye and Robic, 2007). This polymer is composed of disaccharide repeating units, named ulvanobiuronic acids and ulvanobioses. The ulvanobiuronic acids consist of β-D-glucuronic acid or α-L-iduronic acid (1,4)-linked to α-L-rhamnose 3-sulfate (Rha3S), whereas ulvanobioses occur when uronic acids are replaced by β-D-xylose that can be sulphated at position 2 (Kidgell et al., 2019; Lahaye and Robic, 2007).

Although green macroalgae are commonly cultured worldwide (FAO, 2018) due to their high growth rates in diverse geo-climatic conditions (Kidgell et al., 2019), their incorporation in the diets of monogastric animals is limited by the anti-nutritional effects resulting from the recalcitrant nature of their cell wall. The cell wall traps valuable nutrients, leading to a decrease of feed digestion and absorption efficiency (Øverland et al., 2019). Mechanical methods are normally used when adding macroalgae as a feed ingredient or additive (Makkar et al., 2016), but these methods are less selective and sustainable than enzymatic processing (Demuez et al., 2015).

Carbohydrate-Active enZymes (CAZymes) (i.e. pectinases, cellulases (Postma et al., 2018) and carbohydrase mixtures (Batista et al., 2020; Fleurence et al., 1995a)) were shown to be effective on the hydrolysis of green algae biomass with a consequent increase of protein extraction for *U. lactuca* (Postma et al., 2018), *Ulva rigida* (Batista et al., 2020; Fleurence et al., 1995a) and *Ulva rotundata* (Fleurence et al., 1995a). Several reports exist on the use of cellulases (Jmel et al., 2019; Karray et al., 2015; Kim et al., 2011; Trivedi et al., 2013; Yahmed et al., 2016) for the degradation of *Ulva fasciata* (Trivedi et al., 2013), *U. rigida* (Karray et al., 2015), *Chaetomorpha linum* (Yahmed et al., 2016) and *U. lactuca* (Kim et al., 2011); all of them envisaging biotechnological applications (i.e. biogas and bioethanol production). Similarly, carbohydrases were shown to be effective to hydrolyse red (Fleurence et al., 1995b; Harnedy-Rothwell and FitzGerald, 2013; Joubert and Fleurence, 2008; Mæhre et al., 2016) and brown (Habeebullah et al., 2020; Hou et al., 2015; Ravanal et al., 2017; Vanegas et al., 2015) seaweed biomass.

Therefore, CAZymes might be useful to disrupt seaweed cell walls, as previously reported for microalgae (Coelho et al., 2019, 2020). Moreover, some exogenous CAZymes were already found to promote the nutritional value of monogastric diets (Costa et al., 2014; Fernandes et al., 2016), with applications in the poultry and pig feed industry (Ravindran and Son, 2011). The present hypothesis is that CAZymes and sulfatases can, isolated or combined, efficiently disrupt recalcitrant cell walls of *U. lactuca* and improve nutrient bioaccessibility. The breakage of cell walls was evaluated, after enzymatic incubation of macroalgae, through fluorescence microscopy, and analysing the released reducing sugars and oligosaccharides. In addition, the effect of enzyme treatments on releasing algae bioactive and nutritive products was analysed by protein, pigment and fatty acid quantification.

## 2 Materials and methods

### 2.1 Production of macroalgae

Low heat-dried and powdered U. lactuca were purchased from Algolesko Company (Loctudy, Brittany, France). According to the supplier, the algae were cultivated in Asia and biologically certified by Ecocert. A knife mill (Grindomix GM 200, Retsch Gmbh, Haan, Germany) and a woven wire mesh with a diameter of 63 μm (Retsch Gmbh, Haan, Germany) were used to grind and sieve the powdered algae, respectively. Then, algae were stored at - 20 °C before use for in vitro incubations.

### 2.2 High-throughput gene synthesis and cloning, protein expression and purification of recombinant enzymes

A set of 176 CAZymes with potential to degrade the cell wall of macroalgae were selected from a large repertoire, which includes glycoside hydrolases (GH), polysaccharide lyases (PL) and carbohydrate esterases (CE). Moreover, we also selected 23 sulfatases for screening, as it is well known that they play an important role in the degradation of sulphated polysaccharides from macroalgae cell walls (Helbert, 2017). The NZYGene Synthesis kit from Nzytech (Lisbon, Portugal) was used to synthesise in vitro 166 coding genes for the selected enzymes and the remain 33 coding genes were synthesised by Twist Bioscience (San Francisco, CA, USA). The supplementary Table displays the sequence of each enzyme.

Subcloning of the genes into expression vectors, as well as protein expression and purification, were performed as previously described (Coelho et al., 2019, 2020). First, the synthetic genes were optimized for cloning and expression in *Escherichia coli*. Then, the cloning of 166 genes were performed into the bacterial expression vector pHTP1 from Nzytech (Lisbon, Portugal) with the NZYEasy Cloning & Expression kit I (Nzytech, Lisbon, Portugal). The other 33 genes were cloned in pET-29b (+) (Twist Bioscience, San Francisco, CA, USA). The obtained recombinant vectors were submitted to inducible T7 promoter control, but encoding the 199 enzymes fused to an N-terminal His6-tag which allows protein purification by Immobilised Metal Affinity Chromatography (IMAC). All recombinant vectors were sequenced, in order to verify that no mutations occurred during gene synthesis, and were used to transform *E. coli* BL21 (DE3) cells, followed by protein expression and cell harvesting.

The purification of the recombinant enzymes was performed from cell-free extracts by IMAC, using an automated procedure that allows purification of 96 proteins simultaneously (Saez and Vincentelli, 2014). All protein purification steps were automated on a Tecan robot (Tecan, Switzerland), incorporating a vacuum manifold. The purity, homogeneity, and molecular mass of recombinant enzymes were evaluated by 14% SDS-PAGE in comparison with a low molecular weight (LMW) protein marker (Nzytech, Portugal). Protein concentration of enzyme stock solutions was determined spectrophotometrically on NanoDrop 2000c (Thermo Fisher Scientific, Pittsburgh, PA, USA) and varied between 0.13 to 26.7 g/L (Supplementary data Table S1).

### 2.3 Preparation of macroalgae cell suspension

*U. lactuca* was suspended in PBS solution at 20 g/L. This preparation included algae pre-wash, centrifugation and re-suspension, according to the methods reported for microalgae (Coelho et al., 2019).

### 2.4 Algae cell wall degradation by the selected enzyme

Triplicate measurements to assess the degradation of cell wall were completed as previously reported (Coelho et al., 2019), but with one modification; the 24 well-microplate (VWR Chemicals, West Chester, PA, USA) macroalgae incubation with ulvan lyase (20 μg/mL) was stirred overnight at 160 rpm.

### 2.5 Reducing sugar determination

The 3,5-dinitrosalicylic acid (DNSA) method (Miller, 1959) was used for quantification of released reducing sugars, as previously described for microalgae (Coelho et al., 2019). Briefly, 0.6 mL of glucose solutions (0.17 to 1 mg/mL) or in vitro assay supernatants were mixed with 0.6 mL of DNSA reagent. Glucose was used for the standard curve. Afterwards, samples were heated at 100 °C for 15 min followed by 5 min of cooling on ice. The optical density was determined at 570 nm by UV–visible spectroscopy.

### 2.6 Fluorescence microscopic observations

Re-suspension of the pellets obtained from algae and enzyme incubation was done in 1 mL of PBS solution. Calcofluor White, which is a fluorochrome that binds to algae cell wall (Reddy and Fujita, 1991), was acquired from Sigma–Aldrich (St. Louis, MO, USA) and added to the suspension together with a solution of 10% KOH (VWR Chemicals, West Chester, PA, USA) (1:1:1). Microscopic procedures were executed as reported (Coelho et al., 2019) and fluorescence intensity was quantified using Image J software (NIH Image, Bethesda, MD, USA) and expressed as arbitrary units.

### 2.7 Evaluation of ulvan lyase catalytic activity

To analyse PL25 ulvan lyase catalytic activity, ulvan was extracted from *U. lactuca* (Algolesko Company, Loctudy, Brittany, France) according to a previous study (Lahaye et al., 1998), with some modifications. The ground and tamed macroalgae (5 g) was suspended in 1 L of acidic (1.5 mL H_2_SO_4_ at 96%) deionized water and stirred for 30 min. Then, the suspension was filtered through a sterilized cheesecloth, the filtrate was discarded and the ulvan residues were neutralized and extracted with 1 L of 0.1 mol/L NaHCO_3_ with 30 min agitation. The obtained solution was filtered as above and the filtrate was kept (filtrate 1). The residues were extracted with deionized water (1 L) in a boiling water-bath for 1 h and filtered afterwards (filtrate 2). The filtrates 1 and 2 (2 L) were combined and centrifuged (Beckman Coulter Inc., CA, USA) at 5,000 × *g*, for 20 min (10 °C). The obtained supernatant was filtered through a paper filter, adjusted to pH = 6.0 and freeze-dried after storing at − 80 °C. Then, the extract was re-suspended in 200 mL of deionized water (10% of the initial volume) and the soluble starch was hydrolysed via 150 μL of recombinant α-amylase (ID 100) at 2.78 g/L with 30 min of continuous stirring. Afterwards, NaCl was added for a final concentration of 0.1 mol/L, and ulvan was precipitated with 4 vol of ethanol 95%. The precipitate was recovered through centrifugation (Beckman Coulter Inc., CA, USA) at 10,000 × *g*, 10 min, 10 °C and washed twice with 95% ethanol and acetone (Honeywell Riedel-de Haën, Seelze, Germany) before air drying. The crude ulvan extract was resuspended in 500 mL of deionized water and extensively dialyzed. The dialysis was performed against 5 L of deionized water for 24 h with 2 water changes, using SnakeSkin dialysis tubing (Themo Scientific, Waltham, MA, EUA) with a 10 kDa molecular weight cut-off. The retentate was centrifuged (14,000 × *g*, 30 min, 10 °C) and the obtained supernatant was frozen at −80 °C. The ulvan extract was then freeze-dried and stored at − 20 °C.

UV spectroscopy was used to analyse ulvan lyase activity (Foran et al., 2017). A 0.1 mol/L Tris–HCl and 0.2 mol/L NaCl solution (pH = 7.5) was mixed with the extracted ulvan at 1 g/L. In a quartz cuvette, 2.96 mL of the substrate solution was mixed with 40 μL of PL25 ulvan lyase (3.59 mg/mL). A UV–visible spectrophotometer (Pharmacia LKB Ultrospec III spectrophotometer, Gemini, Apeldoorn, Netherlands) was used to continuously record the increase in absorbance at 235 nm during 15 min at 37 °C until linearity was established. A maximum enzyme activity was accomplished after 3 min and was expressed in absorbance units (AU), which consist on the increase in absorbance units per minute.

### 2.8 Thermostability and proteolysis experiments

Thermostability and proteolysis resistance for the PL25 ulvan lyase were evaluated using a protein concentration of 1.47 g/L, and following procedures reported (Coelho et al., 2019). Quantification of the concentration of protein present in the supernatant was measured, in triplicate, using NanoDrop 2000c (Thermo Fisher Scientific, Inc., Pittsburgh, PA, USA) and validated by 14% SDS-PAGE gel electrophoresis in comparison with low molecular weight (LMW) protein marker (Nzytech, Portugal). The evaluation of proteolysis resistance was done as previously described (Coelho et al., 2019). An incubation of ulvan lyase with porcine pancreatin (VWR Chemicals, West Chester, PA, USA) or PBS solution was performed. Then, samples were analysed by 14% SDS-PAGE gels and proteolysis was confirmed through visualization of distinct molecular weight fragments.

### 2.9 Determination of mono- and oligosaccharides

Mono- and oligosaccharide profiles in the supernatants obtained from *U. lactuca* incubation with control and ulvan lyase treatments were determined by HPLC, as described in a previous protocol (Coelho et al., 2019). Glucose was used as the standard to quantify total oligosaccharides, and equivalent moles of released glucose per 100 g of macroalgae was the unit used to express the results.

### 2.10 Protein determination

The nitrogen content in freeze-dried supernatant and residue fractions obtained from *U. lactuca* incubation with the control and ulvan lyase treatment was determined by the Kjeldahl method (984.13) (AOAC, 2000). The formula N × 4.92 was used to calculate CP (Lourenço et al., 2002).

### 2.11 Pigment determination

The quantification of chlorophyll *a* and *b*, total carotenoids and pheophytins was performed as previously reported (Hynstova et al., 2017) with slight modifications (Coelho et al., 2019). These pigment contents were determined, after control and ulvan lyase treatment, in the supernatant and residue fractions obtained from the suspension of *U. lactuca*.

### 2.12 Fatty acid composition evaluation

Fatty acids were extracted, as described for microalgae (Coelho et al., 2019), from freeze-dried supernatants and residue fractions of *U. lactuca* submitted to control and ulvan lyase treatments. For the esterification of fatty acids to methyl esters (FAME), an acidic catalysis procedure was done as previously reported (Cardoso et al., 2017). However, 5 mL of 1.25 mol/L acetyl chloride-methanol solution (Sigma–Aldrich, St. Louis, MO, USA) was used per sample. Nonadecanoic acid (19:0) was used as internal standard for the quantification of total FAME. The analysis of FAME was performed according to procedures already reported (Coelho et al., 2019) and expressed as the percentage of total identified fatty acids.

### 2.13 Statistical analysis

For data analysis in triplicate, the Generalised Linear Mixed (GLM) model of SAS software package was used (version 9.4; SAS Institute, Cary, NC, USA), but for thermostability experiment data, the MIXED procedure of SAS was applied. The SEM is presented as error bars on figures. Results are reported as mean and SEM, and differences were considered significant at *P* < 0.05.

## 3 Results

### 3.1 Enzyme screening for the degradation of *U. lactuca* cell wall

Individual CAZymes and sulfatases (Supplementary data Table S1) were incubated with the suspension of macroalgae to evaluate their capacity to disrupt the cell wall of *U. lactuca*. Most of the enzymes could not break down algae biomass, but 10 enzymes (ID 6, 28, 70, 79, 120, 126, 143, 165, 172 and 183) were able to disrupt *U. lactuca* cell wall, as presented in Table 1. This capability was evaluated by measuring the reducing sugars released using the DNSA method, and the reduction in fluorescence intensity from Calcofluor White stained cell walls using microscopy. These data are shown in Table 1 and displayed as 2 qualitative scales. The first scale measures the release of reducing sugars (g/L): −, 0.00 to 0.37; +, 0.37 to 0.96; ++, 0.96 to 1.73; +++, 1.73 to 3.39; ++++, >3.39, and the second scale is related to the reduction in fluorescence intensity (%): −, 0.00 to 9.50; +, 9.50 to 22.5; ++, 22.5 to 35.4; +++, 35.4 to 40.2; ++++, >40.2. Incubation with the ID 6 enzyme (cellulase) resulted in low levels of reducing sugar released (avg. 0.36 g/L), and only a small decrease in cell wall fluorescence intensity (17.8%). However, this enzyme was selected due to its ability to hydrolyse (1,4)-β-D-glycosidic linkages present in cellulose (Li et al., 2009), which is one of the main constituents of green seaweed cell walls (Lahaye and Robic, 2007).

**Table 1:** Screening of the most active CAZymes and sulfatase on degrading *Ulva lactuca* cell wall^1^.

ID	Name	Category	EC no.	Major substrate	Reducing sugars released scale^2^	Decreased fluorescence intensity scale^3^
6	Cellulase (CelL73)	Cellulases	3.2.1.4	1,3-1,4-β-glucans and soluble 1,4-β-glucans; cellulose	–	+
28	Cellobiohydrolase (CbhA; Cthe0413)	Cellobiohydrolases	3.2.1.91	Amorphous and crystalline cellulose	+	+
70	Endo-β-agarase	Agarases	3.2.1.81	1,4-β-D-galactosidic linkages in agarose	++	+
79	Putative arylsulfatase 2	Arylsulfatases	3.1.6.1	Phenol sulfate	++++	++
120	Endo-1,4-β-xylanase	Xylanases	3.2.1.8	1,4-β-D-xylosidic linkages in xylan	+	++
126	α-Mannosidase	Mannosidases	3.2.1.24	1,2-linked α-D-mannose residues	+	++
143	Alginate lyase	Alginate lyases	4.2.2.3	Alginate	+++	++++
165	Fucanase	Fucosidases	3.2.1.212	(1,4)-α-L-fucoside linkages in fucan	+	+++
172	Ulvan lyase (hypothetical)	Ulvan lyases	4.2.2.-	Ulvan	++++ (4.54 g/L)^4^	++++ (41.7%)^5^
183	Pectate lyase	Pectate lyases	4.2.2.2	(1,4)-α-D-galacturonan from pectate	++	+

CAZymes = carbohydrate-active enzymes; EC = enzyme commission.

1 The column headings are project identification no. (ID), enzyme name, enzyme category, EC no., major substrate and reducing sugar and fluorescence intensity qualitative scales, respectively.

2 Reducing sugars released qualitative scale (g/L): −, 0.00 <0.37; +, 0.37 <0.96; ++, 0.96 <1.73; +++, 1.73 <3.39; ++++, >3.39.

3 Decrease of fluorescence intensity qualitative scale (%): −, 0.00 <9.50; +, 9.50 <22.5; ++, 22.5 <35.4; +++, 35.4 <40.2; ++++, >40.2.

4 Numeric value of released reducing sugars for the most active enzyme on cell wall degradation, ulvan lyase (ID 172).

5 Numeric value of decreased fluorescence intensity for the most active enzyme on cell wall degradation, ulvan lyase (ID 172).

### 3.2 Most active enzyme selection and evaluation of synergistic effects

For the evaluation of additive or synergistic actions between enzymes, a comparison between a mixture of the 10 enzymes selected in the initial screening (Table 1) and a mixture of 3 enzymes (ID 120, 143 and 172) was established. The latter 3 enzymes were selected based on their individual activity on cell wall degradation, producing bacteria, thermostability and substrate. Xylanase ID 120 was isolated from a marine halophilic and hyperthermophilic bacterium (*Thermotoga maritima* MSBS8) (Nelson et al., 1999). Although its specific thermostability has not yet been characterized, a similar enzyme isolated from the same species was shown to be thermoresistant, with an optimum temperature of 90 °C (pH = 6.14) (Zhengqiang et al., 2001). In addition, β-D-xylose, a major constituent of green algae cell wall, can be hydrolysed by enzyme ID 120, which displays activity towards 1,4-β-D-xylosidic linkages in xylans (Kidgell et al., 2019; Lahaye and Robic, 2007). Alginate lyase ID 143, member of polysaccharide lyases family 7 (PL7), was isolated from a marine halophilic bacterium (*Zobellia* galactanivorans DsijT) (Thomas et al., 2012), and belongs to the Flavobacteriaceae family, which includes bacteria that are known as very active on the degradation of algae polysaccharides (Williams et al., 2013). The main substrate of enzyme ID 143 is alginate, which, although only present in brown algae cell walls, is composed of uronic acid units (Deniaud-Bouet et al., 2014), like ulvan (Lahaye and Robic, 2007). Enzyme ID 172 is an ulvan lyase isolated from a marine halophilic bacterium (*Arenitalea lutea*) which, at the time of selection, did not have its activity confirmed. Nonetheless, it shares high homology (52% of identity) with a confirmed ulvan lyase (PL25) isolated from *Pseudoalteromonas* sp. (strain PLSV_3936), showing conservation of all catalytic and main substrate binding residues (PDB: 5UAS, GenBank: WP_036580476.1) (Ulaganathan et al., 2017). The main substrate of enzyme ID 172 is ulvan, which is one of the main constituents of green macroalgae cell walls (Lahaye and Robic, 2007).

The 10-enzyme mixture promoted the release of 4.55 g/L reducing sugars, which was not significantly different (*P* = 0.051) from the value obtained with the 3-enzyme mixture (4.84 g/L). Afterwards, a comparison between the 3-enzyme mixture and the activities of the individual enzymes in its composition was established. A total of 4.84 g/L of released reducing sugars was found with the mixture, which was higher (*P* = 0.011) than that observed with enzyme ID 172 alone (4.44 g/L); although only differing by 0.4 g/L. Conversely, other enzymes demonstrated a highly significant inferior release of reducing sugars (0.58 g/L for enzyme ID 120, and 2.89 g/L for enzyme ID 143) than that of the mixture (*P* < 0.001). Overall, no significant (*P* > 0.05) additive or synergistic effects were found among enzymes.

The released reducing sugar ratios were as follows: ulvan lyase vs. 3-enzyme mixture = 91.7%; ulvan lyase vs. xylanase = 765.5%; and ulvan lyase vs. alginate lyase = 153.6%. These results show that ulvan lyase ID 172 was the most promising enzyme for the disruption of *U. lactuca* cell walls.

### 3.3 Disruption of *U. lactuca* cell wall by ulvan lyase

**Fig. 1:**
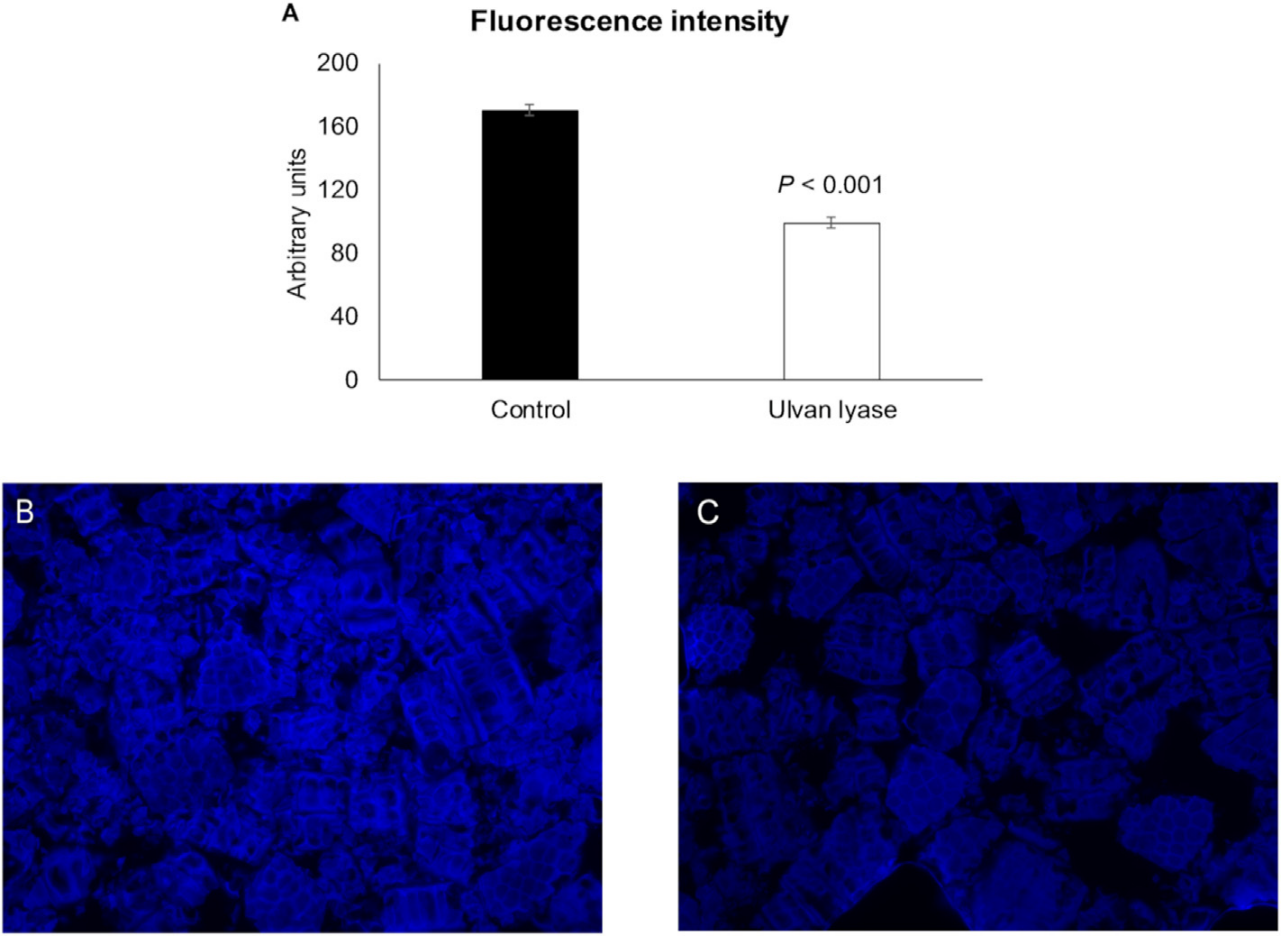
Effect of ulvan lyase (ID 172) on fluorescence intensity of *Ulva lactuca* cell walls: (A) fluorescence intensity derived from Calcofluor White staining for control and ulvan lyase treatment, (B and C) fluorescence images ( × 400) of *U. lactuca* suspension stained with Calcofluor White for control and ulvan lyase treatment, respectively. Mean values are based on 3 replicates per treatment.

The amount of liberated reducing sugars and the reduction of cell wall fluorescence intensity, after incubation of *U. lactuca* with ulvan lyase (ID 172; Provisional Patent No. 20211000002116, INPI, Portugal) are shown in Table 1. Fluorescence intensity results are also presented in Fig. 1A, B, and C. A highly significant increase (*P* < 0.001) of released reducing sugars (4.54 g/L) and reduction (*P* < 0.001) of fluorescence intensity (170.5 to 99.4 arbitrary units; 41.7%) with the ulvan lyase treatment was found in comparison with the control.

### 3.4 Evaluation of ulvan lyase activity, thermostability and proteolysis resistance

**Fig. 2:**
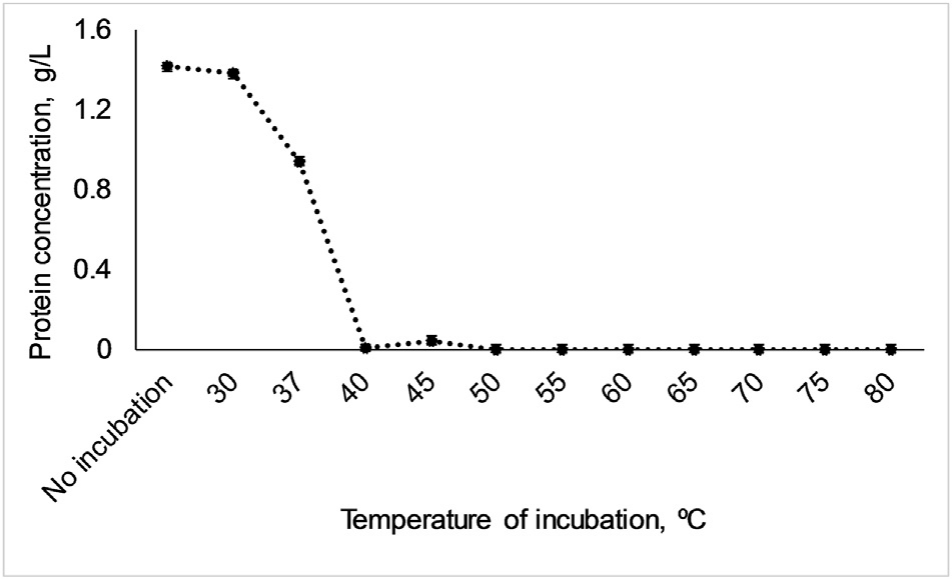
Thermostability of ulvan lyase (ID 172) at different temperatures and for the control without incubation. Values are measured in triplicate.

**Fig. 3:**
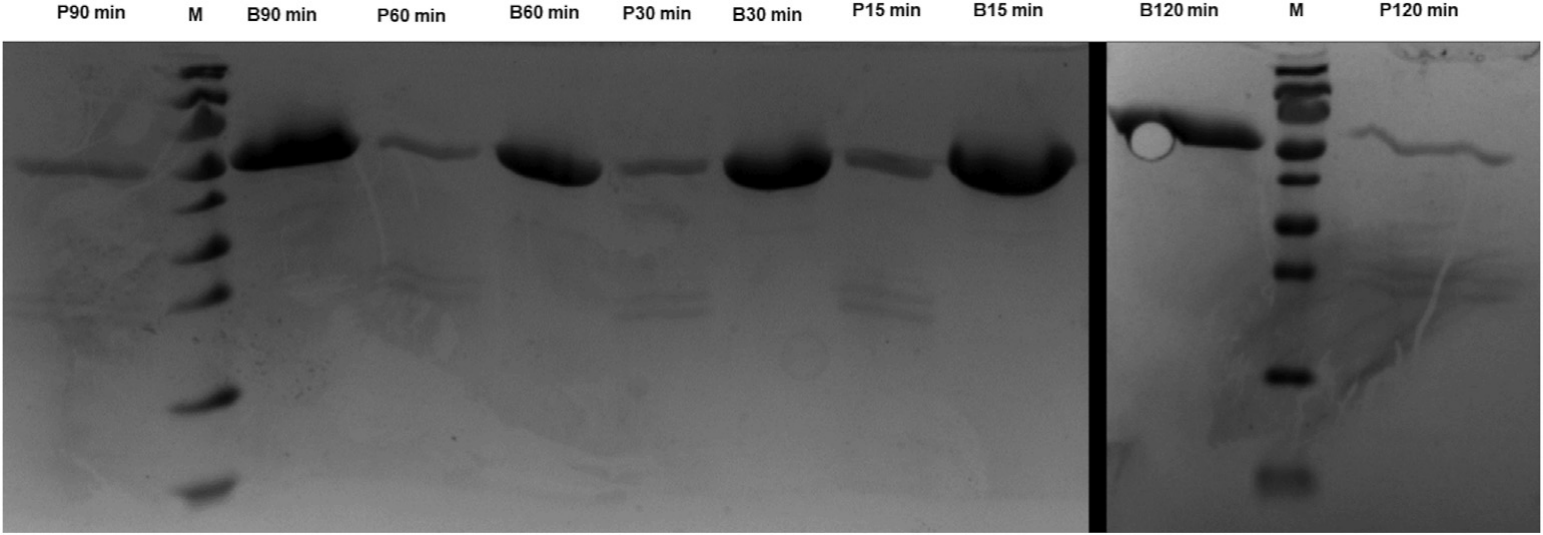
Electrophoresis on SDS-PAGE in 14% (wt/vol) acrylamide gels displaying the fragment bands of ulvan lyase (ID 172) (1.47 g/L) after proteolytic action of pancreatin (final concentration of 2.5 g/L). B = blank, P = purified protein submitted to hydrolysis by pancreatin, M = protein marker.

The enzymatic activity of PL25 ulvan lyase against the substrate ulvan was determined at pH 7.5 and 37 °C, using UV–visible spectroscopy and following procedures adapted from a previous report (Foran et al., 2017). The activity was 1.07 ± 0.027 AU/min at 253 nm. The thermostability of the enzyme was evaluated through subjecting ulvan lyase to different temperatures. Fig. 2 shows the soluble protein concentrations after incubation with a set of temperatures ranging from 30 to 80 °C. The enzyme maintained its stability up to 30 °C, but stability was decreased (*P* < 0.001) by 1.46-fold between 30 °C and 37 °C (1.38 to 0.94 g/L of soluble protein, respectively). Thus, the enzyme was partially stable at 37 °C, which is the normal internal temperature of mammals. However, soluble protein concentrations decline abruptly between 37 and 40 °C, with only residual amounts (0.03 g/L) of the enzyme found between 40 and 45 °C, and complete degradation observed at 50 °C. Table 2 and Fig. 3 show the proteolytic resistance of ulvan lyase. Results indicate a partial resistance of the enzyme during the assay.

**Table 2:** Resistance to proteolysis for ulvan lyase (ID 172) submitted to pancreatin enzymatic activity.

ID	Time, min^1^				
172	15	30	60	90	120
	+	+	+	+	+

Proteolysis resistance was observed in SDS-PAGE gels: -, no resistance (only bands of protein fragmentation); + partial resistance (bands of protein and fragmentation). Pancreatin at a final concentration 2.5 g/L and ulvan lyase (ID 172) at 1.47 g/L.

1 Incubations at 37 °C during periods of 15, 30, 60, 90 and 120 min.

### 3.5 Impact of ulvan lyase on mono- and oligosaccharides released from the cell wall of *U. lactuca*

**Fig. 4:**
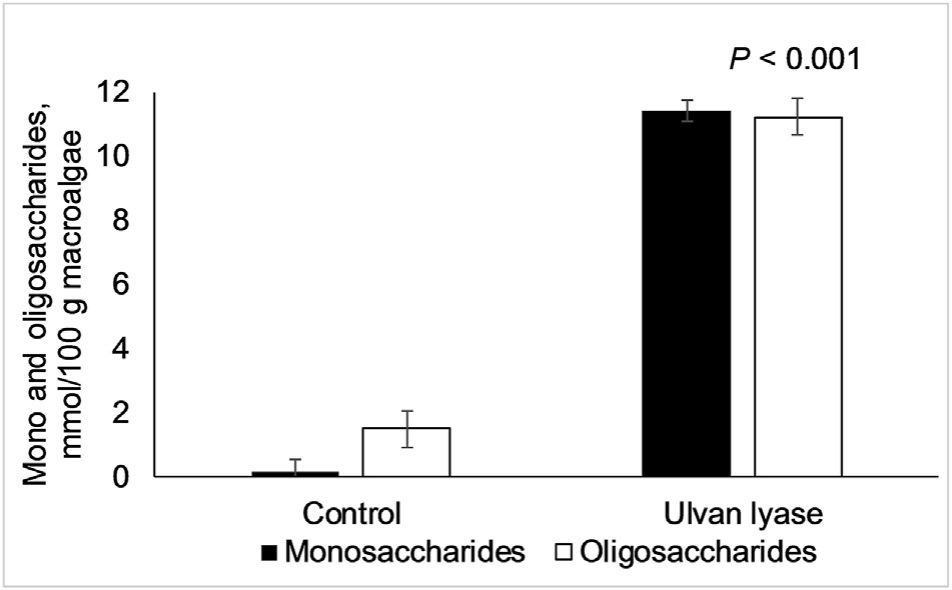
Effect of ulvan lyase (ID 172) treatment on releasing mono- and oligosaccharides from *Ulva lactuca* biomass. Mean values are based on 3 replicates per treatment.

The effect of ulvan lyase treatment on mono- and oligosaccharides released from *U. lactuca* cell wall is shown in Fig. 4. Mono- (*P* = 0.002) and oligosaccharides (*P* < 0.001) concentrations had a highly significant increase with ulvan lyase treatment from 0.19 to 11.4 mmol and from 1.50 to 11.2 mmol/100 g dried algae, respectively, when compared to control. However, released glucose was not detected after the ulvan lyase treatment.

### 3.6 Impact of ulvan lyase on protein and pigment released from the biomass of *U. lactuca*

Table 3 shows the effect of ulvan lyase treatment on pigment and protein concentrations in centrifugation fractions. A similar protein content was produced with both enzyme treatment and control (supernatant with 36.7 and 38.0 mg/g dried alga, and residue with 148 and 164 dried mg/g, respectively); thus, no release (*P* = 0.861) of protein from *U. lactuca* cells to the supernatant was caused by algae incubation with the enzyme. In addition, chlorophyll and carotenoid contents did not differ significantly (*P* > 0.05) between treatments in the supernatant and residue fractions.

**Table 3:** Effect of ulvan lyase (ID 172) on proteins, pigments and fatty acid content (mg/g alga) of supernatant and residue^1^.

Item	Supernatant				Residue			
	Control^2^	UL^3^	SEM	*P*-value	Control^2^	UL^3^	SEM	*P*-value
Total proteins	36.7	38.0	4.68	0.861	148.0	164.1	11.26	0.369
Chlorophyll *a*	0.019^4^	0.013	0.0030	0.187	0.608^5^	0.636	0.0128	0.198
Chlorophyll *b*	0.024^4^	0.018	0.0057	0.474	0.484^5^	0.521	0.0145	0.145
Total chlorophylls	0.043^4^	0.031	0.0086	0.357	1.092^5^	1.157	0.0267	0.160
Total carotenoids	0.011^4^	0.008	0.0008	0.053	0.117^5^	0.115	0.0037	0.766
Total chlorophylls + Total carotenoids	0.054^4^	0.038	0.0091	0.300	1.209^5^	1.272	0.0277	0.180
Total pheophytins	0.198^4^	0.143	0.040	0.386	5.092^5^	5.222	0.1624	0.602

1 Mean values are based on 3 replicates per treatment.

2 Control, 2 mL of resuspended macroalgae (20 g/L solution of PBS).

3 UL, ulvan lyase treatment; 2 mL of macroalgae suspension at 20 g/L plus ulvan lyase (ID 172) at 20 mg/L in PBS solution.

4 Values measured in PBS.

5 Values measured after extraction with acetone.

### 3.7 Impact of ulvan lyase on fatty acids released from the biomass of *U. lactuca*

The fatty acid profile obtained by the algae incubation with PL25 ulvan lyase was assessed to evaluate the enzymatic effect on promoting the release of fatty acids from *U. lactuca* cells to the external environment (data not shown).

The percentage of fatty acids was as follows: saturated fatty acids (SFA) > MUFA > PUFA and n-6 PUFA for supernatant fractions, and SFA > MUFA > PUFA > n-3 PUFA > n-6 PUFA for residue fractions. The sum of fatty acids in the supernatant did not differ significantly (*P* = 0.069) between the ulvan lyase treatment and the control; corresponding to 0.90 and 1.27 mg/g dried alga, respectively. No differences (*P* > 0.05) were found for the percentage of individual fatty acids (% total fatty acids). The sum of fatty acids recovered in the residue did not differ significantly (*P* = 0.294) between ulvan lyase treatment and control, which corresponded to 3.71 and 3.07 mg/g dried alga, respectively. However, the enzyme treatment led to a 2-fold increase (*P* < 0.001) of 18:1c9, from 3.18% to 6.44%, and a 3-fold increase (*P* = 0.030) of 20:1c11, from 0.21% to 0.60%, in comparison to the control.

## 4 Discussion

One-hundred and seventy-six CAZymes and 23 sulfatases were selected for testing the hypothesis that some of them could degrade the intricate *U. lactuca* cell wall and promote nutrient accessibility. In order to produce these enzymes, many procedures were executed in a high-throughput (HTP) platform, which mainly included gene cloning, expression and purification of recombinant proteins. The selection of the 199 recombinant enzymes was done according to the composition of the cell wall polysaccharide matrix found in green macroalgae. This matrix is known to be composed of ulvan, cellulose, xyloglucan, glucuronans and, for some algae species, mannose, galactose and arabinose (Kidgell et al., 2019; Lahaye and Robic, 2007). Additionally, the selection of enzymes also took into account their origin, with the majority being produced by marine halophilic bacteria (121) and a considerable set of enzymes originated from thermophilic bacteria (41).

All the enzymes were individually screened to evaluate their capacity to disrupt the cell wall of *U. lactuca*. The screening was performed by determining the amount of reducing sugars released and the fluorescence intensity of Calcofluor White stained algae cell walls. Afterwards, two combinations of recombinant enzymes, one with 10 enzymes (Table 1) and the other with 3 enzymes (xylanase ID 120, alginate lyase ID 143 and putative ulvan lyase ID 172), were done to test the maximum cell wall degradation in *U. lactuca* suspensions. However, enzymes did not act additively or synergistically and ulvan lyase was, therefore, selected as the most effective in degrading macroalgae cell walls.

Ulvan lyase (ID 172) is produced by a marine bacteria (*A. lutea*) and its amino acid sequence (GenBank accession no. SHI30876) shares 52% identity with that of a well-characterized ulvan lyase from polysaccharide lyases family 25 (PL 25; EC 4.2.2.-) from marine halophilic bacterium Pseudoalteromonas sp. PLSV (PLSV_3936, GenBank accession no. WP_033186995.1) (Ulaganathan et al., 2017). Both enzymes are BNR containing proteins and show complete conservation of the putative catalytic and substrate binding residues, identified by the structure of *Pseudoalteromonas* sp. enzyme in complex with ulvan tetrasaccharide (PDB 5UAS) (Ulaganathan et al., 2017). Therefore, it was inferred that, similarly to ulvan lyase from *Pseudoalteromonas* sp. PLSV, the enzyme with ID 172 is able to depolymerize ulvan into di- and tetrasaccharides of uronic acids, by cleaving glycosidic bond between glucuronic or iduronic acid residues and Rha3S (Gao et al., 2019; Ulaganathan et al., 2017). A recombinant ulvan lyase (ID 166) with the same catalytic mode as that of the enzyme isolated from *Pseudoalteromonas* sp. PLSV, was also tested in the present study, but it led to an inferior numerical decrease of fluorescence intensity compared to the enzyme ID 172 (23% vs. 42%). Therefore, enzyme ID 166 was considered less efficient in degrading seaweed cell wall than enzyme ID 172, although the reducing sugars assay showed no differences (*P* = 0.862) between the two enzymes.

PL25 Ulvan lyase had partial proteolysis resistance and thermostability at up to 37 °C. However, a complete degradation of the enzyme was found between 40 and 50 °C. The thermal stability of ulvan lyases from PL25 family is scarcely reported, with two studies showing optimum activity at 45 °C (Foran et al., 2017) and 50 °C (Gao et al., 2019) for ulvan lyases produced by *Alteromonas* sp., which indicates a higher thermostability of these enzymes than that of enzyme ID 172. Similarly to enzyme ID 172, other ulvan lyases from different families, such as PL24 produced by *Pseudoalteromonas* sp. PLSV (Qin et al., 2018) and PL28 produced by the marine halophilic flavobacterium *Formosa agariphila* (Reisky et al., 2018), were shown to be thermostable only at lower temperatures (35 °C (Qin et al., 2018) and 29.5 °C (Reisky et al., 2018), respectively). The fact that the enzyme ID 172 originated from a mesophilic (instead of a thermophilic) Gram-negative bacterium—*A. lutea*—may explain its poor thermal stability. Nevertheless, *A. lutea* is a marine halophilic organism that belongs to the Flavobacteriaceae family, which includes bacterial species with the ability to utilize a great variety of algal polysaccharides (Williams et al., 2013), including ulvan (Collén et al., 2011; Reisky et al., 2019). Further studies involving genetic engineering processes (Druzhinina and Kubicek, 2017), such as screening AA sequences that would confer thermotolerance to the enzyme ID 172 and site-directed mutagenesis, will be done in order to increase enzymatic thermostability.

The present study is the first to demonstrate the efficiency of reducing sugar release by a PL25 ulvan lyase (4.54 g/L, 227 mg/g dried alga) from green macroalgae cell walls. In fact, other reports have only described the ability of cellulases, which were either commercially acquired (Trivedi et al., 2013) or produced, as enzymatic extracts from *Aspergillus* (Karray et al., 2015; Trivedi et al., 2013), to release reducing sugars from *U. rigida* (7.3 g/L) (Karray et al., 2015), *U. fasciata* (up to 215 mg/g dried alga) (Trivedi et al., 2013) and *C. linum* (220 mg/g dried alga) (Yahmed et al., 2016) biomass, under the context of producing biogas or bioethanol.

Release of reducing sugars by the PL25 ulvan lyase action on *U. lactuca* likely corresponds to the mono- and oligosaccharide content detected by HPLC. Despite this, glucose was not detected in the extracellular medium. Although the selectivity of ulvan lyases against carbohydrates present in the cell wall of green macroalgae has not been studied to date, other reports analysed the amount of soluble carbohydrates (Kim et al., 2011; Postma et al., 2018) released from *U. lactuca* biomass for biorefinery purposes. Postma *et al.* (2018) described a release of total carbohydrates from macroalgae biomass when using a commercial pectinase or cellulase, whereas Kim *et al.* (2011) reported a release of soluble sugars through the action of commercial cellulases.

The reduction of fluorescence intensity from the cell wall of *U. lactuca* (41.7%) stimulated by PL25 ulvan lyase shows that algae cell wall was partially degraded, as previously reported in microalgae (Coelho et al., 2019, 2020). The breakdown of cell wall was probably caused by the action of enzyme ID 172 through endolytic cleavage of ulvan, with a consequent compromise of cell wall rigidity dependent on the flexibility provided by the helix conformation of gel-forming ulvan (Lahaye and Robic, 2007). The increase in absorbance at 235 nm, observed when the enzyme ID 172 acted on the ulvan substrate (1.07 ± 0.027 AU/min), indicates that the enzyme effectively degrades ulvan with a subsequent release of uronic acid oligosaccharides containing C4 – C5 double bonds (Gao et al., 2019; Ulaganathan et al., 2017).

Conversely, PL25 ulvan lyase caused no release of (hydro-) soluble proteins from *U. lactuca* biomass, which might be caused by the presence of hydrocolloidal anionic polysaccharides (i.e.*,* products of ulvan degradation) in the extracellular medium. These polysaccharides could have increased medium viscosity, and thus restricted the methodology and quantification of proteins (Lahaye and Robic, 2007; Li et al., 2015). In fact, an increase of viscosity was observed in ulvan lyase treatment supernatants. This phenomenon was previously suggested to limit protein extraction in a study showing the effects of a cellulase and carbohydrase mixture on *U. rigida* and *U. rotundata* (Fleurence et al., 1995a). Additionally, ulvan lyase treatment did not extracellularly release pigments from the cells of *U. lactuca*. Pigment concentration in the control residue was lower than the amounts (mg/g dried alga) already described for *Ulva* sp. (Eismann et al., 2020) (0.61 vs. for 2.13 chlorophyll *a*, 0.12 vs. 2.93 for total carotenoids), probably due to variations in algae growth and post-harvesting conditions (Eismann et al., 2020). Possibly, PL25 ulvan lyase did not breakdown the thylakoid membrane of chloroplasts, which contain the photosynthetic pigments (Murakami and Packer, 1970), as suggested for microalgae (Coelho et al., 2019, 2020).

Enzyme ID 172 did not extracellularly release fatty acids from algae (supernatant), but it could modify fatty acid profile of the algae biomass (residue fraction), with an increase in the oleic acid (18:1c9) and 20:1c11 fatty acid. Therefore, the current study shows a significant influence of an ulvan lyase (PL25) on the fatty acid profile of a green seaweeds (*U. lactuca*). The 2-fold increase of 18:1c9 in the algae biomass with the ulvan lyase treatment deserves further exploitation, due to the benefits of this fatty acid to hinder human cardiovascular disease (Givens, 2009).

## 5 Conclusions

The results obtained herein show that an individual recombinant PL25 ulvan lyase partially disrupts the cell wall of *U. lactuca* under physiological conditions. This enzymatic ability can release valuable bioactive compounds that were trapped by the recalcitrant structure of algae cell wall, mainly by the main gel-forming polysaccharide of green macroalgae cell wall, the ulvan. Such compounds can be of major importance for the biotechnological and feed industries. Therefore, ulvan lyase from PL25 family can act as a biocatalyst supplement for monogastric animal diets incorporated with *U. lactuca* as a feed ingredient. Future studies are presently being performed by our team to evaluate the efficacy of this recombinant ulvan lyase on stimulating the release of nutritional compounds after its utilization in monogastric diets with *U. lactuca* at high inclusion percentages (up to 15% feed).

## Author contribution

Mónica M. Costa: Methodology, Validation, Formal analysis, Investigation, Writing – original draft, Writing – review & editing. Luís B. Pio: Methodology, Formal analysis, Investigation. Pedro Bule: Methodology, Data curation, Writing – review & editing. Vânia A. Cardoso: Investigation. Marlene Duarte: Investigation. Cristina M. Alfaia: Formal analysis. Diogo F. Coelho: Methodology, Formal analysis. Joana A. Brás: Supervision. Carlos M.G.A. Fontes: Conceptualization, Data curation. José A.M. Prates: Conceptualization, Validation, Writing – review & editing, Supervision, Project administration, Funding acquisition.

## Declaration of competing interest

We declare that we have no financial and personal relationships with other people or organizations that can inappropriately influence our work, and there is no professional or other personal interest of any nature or kind in any product, service and/or company that could be construed as influencing the content of this paper.

## Appendix A Supplementary data

Multimedia Comp 1 Multimedia Comp 1
